# Anxiety, depression, and quality of life in backache patients before and after spinal traction

**DOI:** 10.1186/s41983-018-0048-5

**Published:** 2018-12-29

**Authors:** Amr Said Shalaby, Dina Rifaat el-sharaki, Gelan Mahmoud Salem

**Affiliations:** 1 Department of neuropsychiatry, University of Menoufia, Shibin El koum, Egypt; 2 Department of physical medicine, University of Menoufia, Shibin El koum, Egypt

**Keywords:** Backache, Spinal traction, Psychological status, Quality of life

## Abstract

**Background:**

Chronic pain has a negative impact on quality of life and psychological well-being. The objectives of this study are to investigate the psychological status and quality of life in backache patients before and after spinal traction and reflect how this maneuver is effective in treating backache.

**Methods:**

Forty-seven backache patients completed the hospital anxiety and depression scale (HADS) and Short-Form 36 Health Survey (SF-36) before and after treatment with spinal traction. Forty-eight healthy controls, matched with patients for age and sex, completed the same questionnaires. Pain was assessed before and after the maneuver using a visual analog scale (VAS). Traction was added to patients’ medications which were not enough to control patients’ symptoms and did not change during the course of traction.

**Results:**

Before spinal traction, the mean VAS score was 7 ± 1.36, abnormal levels of anxiety and depression were found in 36.17% and 40.43%, respectively, of patients, and all SF-36 domains of the study population, except for physical functioning, showed mean scores < 50%. After spinal traction, the mean VAS score dropped significantly to 5.44 ± 1.51, abnormal levels of anxiety and depression became 14.9% and 21.3%, and all SF-36 domains improved significantly, with six of the eight domains showing mean scores > 50%. There were significant differences regarding all SF-36 domains, and anxiety and depression scores between patients and controls, in favor of controls, before traction. These differences disappeared after spinal traction.

**Conclusion:**

Pain, psychological status, and quality of life improved when spinal traction was added to medications reflecting its efficacy for patients with backache.

## Introduction

Chronic back pain is a common health problem resulting in significant personal, social, and occupational impairment; role disability; and health care utilization [1].

Reduced quality of life is common among patients suffering from chronic low back pain [2]. Anxiety and depression are the two most common forms of psychological disturbances seen in patients with chronic backache [3]. This combination—depression, anxiety, and pain—is associated with worse clinical outcomes than each condition alone [4].

Although there is conflicting evidence for its efficacy for patients with low back pain, there is preliminary evidence that lumbar traction produce positive results in nerve root compression symptoms. Regarding degenerative and discogenic pain, data are debatable [5]. By using traction, therapists aim to achieve a separation of the joint surfaces and a decrease of disc protrusion, resulting in removal of the compression in the surrounding tissues [6].

The present study aims to assess the psychological status and quality of life in a group of backache patients before and after spinal traction, to reflect how this maneuver is effective in treating their conditions and whether longer duration of treatment is more beneficial, and, finally, to detect correlations and associations among variables studied.

## Study design

### Participants

This prospective study was carried out on 47 recruited patients seeking treatment at the Physical Medicine, Rheumatology and Rehabilitation Department, Menoufiya University Hospitals, in the period between January and July 2015.

Two groups of patients were included in the present study: the first being those with the diagnosis of prolapsed lumbar disc and the second with lumbar spondylosis “osteoarthritis” diagnosis. Patients were diagnosed to have lumbar spondylosis based on clinical examination and plain x-ray lumbosacral spine. Patients with prolapsed lumbar disc were diagnosed on the basis of clinical examination and MRI lumbosacral spine. Patients showed only some improvement on medical treatment, NSAI drugs, muscle relaxants, and drugs for neuropathic pain, which was continued and not changed during the course of spinal traction.

In addition to non-consenting patients, patients with the following conditions were excluded from the present study: ligamentous instability, osteomyelitis, diskitis, primary or metastatic spinal cord tumor, severe osteoporosis, myelopathy, fibromyalgia, or untreated hypertension. Patients currently treated for psychiatric disorders, or those who had past history of psychiatric disorders, were also excluded.

Forty-eight health controls, matched with patients for age and sex, were recruited from employees working at the Faculty of Medicine, Menoufia University, and its hospitals.

## Methods

### Interviewing questionnaire

For recording socio-demographic data, medical history, results of clinical examination, and radiological investigations (plain x-ray and MRI), an interviewing questionnaire was used.

### Visual analog scale (VAS) [7]

The intensity of the pain was measured using a visual analog scale (VAS) in which the intensity of pain was classified as 0 to 10, as follows: 0–1 = no pain, 2–3 = mild pain, 4–5 = moderate pain, 6–7 = intense pain, and 8–10 = extreme pain.

### Hospital anxiety and depression scale

Depressive and anxiety symptoms were assessed by an Arabic version of Hospital Anxiety and Depression scale [8]. HAD scale is a questionnaire with the dimensions anxiety and depression containing 14 items, 7 referring to anxiety and 7 to depression, with a cut-off point of 8 for anxiety and 9 for depression. All items refer exclusively to the emotional state and do not reflect somatic symptoms.

### Short-Form 36 Health Survey (SF-36)

Health-related quality of life was assessed by applying the Arabic version of the Short-Form 36 Health Survey (SF-36) [9]. This is a generic instrument composed of 36 items that evaluate the following: physical functioning (the ability to take care of oneself and to perform routine daily activities), role limitations due to physical health (the impact of physical health in performing activities), bodily pain (level of pain experienced while performing routine daily activities), general health perceptions (how the individual perceives his/her health), vitality (energy and fatigue), social functioning (impact of physical conditions on his/her social life), role limitations due to emotional problems (the extent to which emotional problems interfere in routine daily activities), and mental health (effect of mood on his/her life). To analyze the eight domains of the scale, a score ranging from 0 (most affected) to 100 (not affected) is used.

### Lumbar traction apparatus

ELTRAC 471, ENRAF-NONIUS, Netherlands, is a microcomputer-controlled unit for continuous and intermittent spinal traction. In the ELTRAC 471, the traction force is controlled and monitored by the built-in microprocessor. The tension in the cord is measured electronically and compared with the set value.

A further advantage of the electronic traction control is that the speed of applying and releasing the traction force can be adjusted. In addition to the electronic safety system, the unit is also provided with a mechanical traction limiting system and the patient can stop the switch which enables the patient to terminate the session.

#### Techniques of application

Patient who had undergone lumbar traction was exposed to a superficial heat physical therapy at the lumbosacral area by using infrared therapy for a 10-min duration. This was immediately followed by lumbar traction session. The duration of the lumbar traction session was 20 min.

The patient was lying down in supine position. The mode of application of lumbar traction was intermittent application determined by patient’s body weight and gradually increased in further sessions according to patient’s tolerance. Patients were divided into two groups, the first group received 4 weeks of spinal traction (28 patients 59.5%) and the second received more than 4 weeks and up to 12 weeks of treatment (19 patients 40.5%).

Controls filled the sociodemographic sheet, and both hospital anxiety and depression scale (HADS) and Short-Form 36 Health Survey.

### Statistical analysis

All analyses were done using the software SPSS 20. IBM Corp. Armonk, NY, USA. As variables were not normally distributed, Wilcoxon signed ranks test was used to compare the patients’ results before and after spinal traction, and Spearman correlation test to detect any correlations between tested variables. Chi-square test and Fischer’s exact test were also used to detect any associations between different variable improvements after spinal traction. Statistically significant findings were determined by a two-tailed *p* value < 0.05. The Man-Whitney test was used to compare the controls’ and patients’ results once before the spinal traction and another the time after.

## Results

### Demographics

#### Patients

The present study included 47 patients. They were 29 females (59.6%) and 18 males (40.4%), and the mean age was 42.55 ± 13.13. Patients were presented with two main diagnoses, prolapsed lumbar disc (33 patients, 70.2%), and spondylosis (14 patients, 29.8%). The duration of illness ranged from few months and up to 10 years with mean ± SD 24.28 ± 27.84, and traction duration ranged from 4 to 12 weeks.

#### Controls

Forty-eight controls were included in the study. They were 26 females (54.16%) and 22 males (45.84%), and the mean age was 42.55 ± 13.13.

#### VAS

Only six patients scored below 6, mild to moderate pain, while all other patients’ scores reflected intense and extreme pain. Before starting spinal traction, VAS score ranged from 3 to 9 with mean ± SD 7 ± 1.36. Following lumbar traction, VAS score ranged from 2 to 9 with mean ± SD 5.44 ± 1.51.

#### HADS

Before starting spinal traction, abnormal levels of anxiety and depression, HADS 11–21, were found in 36.17% and 40.43% of patients, while after spinal traction these percentages became 14.9% and 21.3%.

Before spinal traction, anxiety and depression scores were 9.57 ± 3.57 and 9.68 ± 3.26, respectively. After spinal traction, the scores became 7.57 ± 3.15 and 7.96 ± 2.77. No differences were found between male and female patients regarding anxiety or depression before spinal traction.

#### Short-Form 36 Health Survey (SF-36)

Table 1 shows the scores of the eight domains of the SF-36 Health Survey before and after spinal traction. The scores of role limitation due to physical health and role limitation due to emotional health domains showed the highest increase among other SF-36 Health Survey domains.

**Table 1:** SF-36 scores before and after spinal traction

SF-36 domains	Before spinal traction	After spinal traction	Wilcoxon signed rank test	*P*
Physical functioning	56.81 ± 25.589	65.49 ± 18.733	− 3.116	0.0001
Role limitations due to physical health	7.60 ± 22.036	35.74 ± 36.296	− 4.45	0.0001
Bodily pain	38.10 ± 16.877	55.27 ± 15.753	− 5.419	0.0001
General health perceptions	47.27 ± 15.459	51.04 ± 16.253	− 3.093	0.0001
Vitality	37.38 ± 15.560	50.94 ± 13.940	− 5.06	0.0001
Social functioning	43.99 ± 22.243	57.54 ± 17.450	− 4.571	0.0001
Role limitations due to emotional problems	13.38 ± 32.531	40.15 ± 42.647	− 3.718	0.0001
Mental health	49.77 ± 17.236	57.66 ± 14.980	− 3.749	0.0001

*SF-36* Short-Form 36 Health Survey

Patients who underwent spinal traction for more than 4 weeks did not show more improvement than patients who received 4 weeks of treatment, except for improvement in SF-36 (*z* test = − 2.98 *p* = 0.003).

Table 1 shows a comparison between controls and backache patients, before and after spinal traction, regarding HADS and SF-36 scores. There were significant differences regarding all SF-36 domains and HADS anxiety and depression scores between patients and controls, in favor of controls, before spinal traction. After the maneuver and with the improvements of patients’ scores, all these differences disappeared.

Table 2 shows the correlations that were found statistically significant among scores of VAS, anxiety and depression of HADS, and the eight domains of SF-36 health survey.

**Table 2:** Correlation among VAS, HADS, and SF-36 domains scores before spinal traction

	VAS	SF-36 bodily pain	HADS-D	HADS-A
Physical functioning	X	Rho 0.347 *p* = 0.017		Rho − 0.317 *p* = 0.03
Role limitations due to physical health	X	X	X	X
Bodily pain	X	X	X	X
General health perceptions	X	Rho 0.408 *p* = 0.004	Rho − 0.365 *p* = 0.012	
Vitality	X	Rho 0.467 *p* = 0.001	Rho − 0.334 *p* = 0.022	Rho − 0.311 *p* = 0.033
Social functioning	X	Rho 0.538 *p* < 0.0001	X	Rho − 0.372 *p* = 0.01
Role limitations due to emotional problems	X	Rho 0.289 *p* = 0.049	X	X
Mental health	X	X	Rho − 0.45 *p* = 0.002	Rho − 0.457 *p* = 0.001
VAS	X	X	X	X
HADS-D	X	X	X	X
HADS-A	X	X	X	X

*X* no correlation found, *VAS* visual analog scale, *HADS* hospital anxiety and depression scale, *SF-36* Short-Form 36 Health Survey

Table 3 shows the associations found, using the chi-square test and Fischer exact test, among the improvements of VAS, HADS, and SF-36 domains after spinal traction. Improvements were determined based on the decrease of VAS, anxiety and depression scores, and the increase of SF-36 domains scores.

**Table 3:** associations among score improvements of VAS, HADS, and SF-36 domains by chi-square test

	VAS Improved 37 Not 10	SF-36 bodily pain Improved 39 Not 8	HADS-D Improved 28 Not 19	HADS-A Improved 32 Not 15
Role limitations due to physical health Improved 25 Not 22	X	X	X	X
Bodily pain Improved 8 Not 39	X	Fisher’s exact test 6.28 *p* = 0.012	Chi-square 5.98 *p* = 0.014	X
General health perceptions Improved 21 Not 16	X	X	Fisher’s exact test 4.68 *p* = 0.047	X
Vitality Improved 28 Not 19	X	X	X	X
Social functioning Improved 30 Not 17	X	X	Chi-square 6.19 *p* = 0.013	X
Role limitations due to emotional problems Improved 19 Not 28	X	Fisher’s exact test 10.76 *p* = 0.002	X	X
Mental health Improved 27 Not 20	X	Fisher’s exact test 6.4 *p* = 0.015	Chi-square 8.03 *p* = 0.005	Chi-square 6.7 *p* < 0.01
Physical functioning Improved 26 Not 21	X	X	Chi-square 19.56 *p* < 0.0001	X
VAS Improved 37 Not 10	X	Chi-square 13.01 *p* < 0.0001	Chi-square 8.73 *p* = 0.003	Chi-square 5.24 *p* = 0.022
HADS-D Improved 28 Not 19	X	X	X	X
HADS-A Improved 32 Not 15	X	Fisher’s exact test 4.68 *p* = 0.047	X	Chi-square 19.56 *p* < 0.0001

*X* no association found, *VAS* visual analog scale, *HADS* hospital anxiety and depression scale, *SF-36* Short-Form 36 Health Survey

## Discussion

With chronic pain, it is extremely difficult for individuals to perform physical activities. In addition, chronic pain is usually co-morbid with psychiatric disorders. Both conditions are often inadequately treated and result in substantial disability and reduced health-related quality of life [5].

In the present study, the mean VAS scores of the study population dropped significantly from 7 ± 1.36 before starting spinal traction to 5.44 ± 1.51 after finishing the treatment course.

Beattie and colleagues found that 84.4% of patients with low back pain, and with evidence of a degenerative and/or herniated intervertebral disc, reported significantly improved pain scores after 16 to 24 visits of prone traction at discharge and at 30 days and 180 days post-discharge [10].

On the other hand, a systematic review by Clarke and colleagues [11] revealed that intermittent or continuous mechanical traction as a single treatment for low back pain cannot be recommended for heterogeneous groups of patients suffering from low back pain with or without sciatica.

In the present study, spinal traction was added to medical treatment which alone was not enough to control patients’ symptoms and which was not changed during traction. This combination of spinal traction and medications may explain the better response in the present study when compared with other studies.

Considering the two different diagnoses included in the present study, patients with lumbar prolapsed disc showed a significant decrease of VAS scores from 7.18 ± 1.16 before traction to 5.25 ± 1.28 after traction. At the same time and although VAS scores for patients with the diagnosis of spondylosis decreased after traction from 6.79 ± 1.76 to 5.89 ± 1.95, this decrease was not statistically significant. On the contrary, the pain domain of SF-36 showed significant improvement in both patient groups after traction. As SF-36 pain domain assesses how pain limits daily activities, it may be more reliable to consider its results than VAS which assesses pain subjectively and in a general manner.

Patients who underwent spinal traction for more than 4 weeks showed more pain improvement on SF-36 than patients with 4 weeks or less durations of treatment. Again, this was not the case when considering pain scores measured by VAS.

In the present study, abnormal levels of anxiety and depression were found, respectively, in 36.17% and 40.43% (HADS 11–21) of patients complaining of backache. If adding patients who scored 8–10, borderline abnormal, the percentages will rise to 76.59 and 78.72%, respectively.

A Pakistani study investigated the prevalence of anxiety and depression in 140 patients with chronic low back pain using HADS revealed that 16.4% and 12.1% of the patient scores for anxiety and depression, respectively, were abnormal (HADS 11–21). A study of 70 German patients with back pain reported 36% of patients with abnormal anxiety (HADS-A > 10) and 29% with abnormal depression (HADS-D > 8) [4]. In Brazil, Castro and colleagues found that 70% and 60% of patients with low back pain had anxiety and depression symptoms, respectively, using HDAS [5].

According to Kayhan and his colleagues, mood and anxiety disorders were more commonly seen in patients with lumbar disc herniation than in control subjects [12]. Also, the study of Von Korff and colleagues. [13] showed that chronic back pain was significantly associated with mood, anxiety, and also alcohol abuse and dependence disorders.

In the present study and after spinal traction, anxiety and depression scores measured by HADS dropped significantly from 9.57 ± 3.57 and 9.68 ± 3.26 to 7.57 ± 3.15 and 7.96 ± 2.77, respectively. Following traction, the percentages of patients who scored (11–21) on HADS dropped from 36.17% and 40.43% to 14.9% and 21.3% for anxiety and depression, respectively.

As shown in Table 1, before spinal traction, all SF-36 domains of the study population, except for physical functioning, showed mean scores < 50%. After finishing the treatment course, all SF-36 domains improved significantly, with six of the eight domains showing mean scores > 50%.

According to Bentsen SB and colleagues [3], reduced quality of life is common for patients suffering from chronic low back pain (CLBP) and the goal of treatment is to optimize patients’ quality of life in terms of less pain and better functioning. In the study performed by Castro and his colleagues on anxiety, depression, and quality of life in patients with low back pain [5], they found that the eight domains of SF-36 showed mean scores ≤ 50%. (Table 4).

**Table 4:** comparison of SF-36 results with other studies

SF-36 domains	Present study (No. 47)	Castro MMC et al., 2011 (No. 400)	Bentsen et al., 2008 (No. 25)
Physical functioning	56.81 ± 25.589	36.2 ± 22.6	44.2 ± 19.2
Role limitations due to physical health	7.60 ± 22.036	17.8 ± 27.9	6 ± 22
Bodily pain	38.10 ± 16.877	31.8 ± 17.4	23.9 ± 15.4
General health perceptions	47.27 ± 15.459	44.4 ± 21.8	64.4 ± 21.2
Vitality	37.38 ± 15.560	41.3 ± 22.6	30.8 ± 15
Social functioning	43.99 ± 22.243	49.5 ± 27.9	40.5 ± 21.7
Role limitations due to emotional problems	13.38 ± 32.531	30.0 ± 36.6	57.3 ± 45.7
Mental health	49.77 ± 17.236	48.7 ± 23.2	64.8 ± 19.8

*SF-36* Short-Form 36 Health Survey

Despite that the scores of VAS and HADS for both anxiety and depression significantly decreased after spinal traction, no correlations were found between these three variables before traction. Comparing patients’ results before and after traction, chi-square test did not show any significant association between the improvement of VAS scores and any other improvements. Again, and at the same time, chi-square test showed an association, although weak, between the improvement of SF-36 bodily pain domain and depression (*p* = 0.047).

According to Hedda van’t Land and colleagues, the risk of developing a mood or anxiety disorder was significantly higher among subjects with chronic back pain on both cross-sectional and longitudinal models [1].

Castro and his colleagues found that patients with severe or extreme pain had a higher frequency of anxiety than those with mild to moderate pain. However, the frequency of depression did not reach statistical significance when both groups were compared. In our study, only six patients scored below 6, mild to moderate pain according to VAS, while all other patients’ scores reflected intense and extreme pain, making it difficult to compare between the two groups [5].

Psychological mechanisms have been suggested to explore the relationship between pain and both anxiety and depression. Research findings suggest that affective and cognitive variables influence pain and disability [14]. According to Picavet and his colleagues, a high level of pain catastrophizing or a high level of kinesiophobia, fear of movement/(re)injury, increases the risk of future chronic low back pain and disability [15]. In the present study and despite no correlation was found between each of anxiety and depression scores and pain scores before traction, negative correlation between anxiety scores and each of SF-36 physical functioning and social functioning domains were found. This may emphasize the role of kinesiophobia in limiting patients’ daily activity and affecting their quality of life. Which agrees with this is that role limitation due to emotional health was one of two SF-36 Health Survey domains that showed highest increase after spinal traction.

The study of van’t Land and his colleagues provided empirical support for two hypotheses, the consequence hypothesis (chronic back pain precedes the development of psychiatric disorders) and the antecedent hypothesis (anxiety disorders precede the development of chronic back pain) [1]. According to the same study, another hypothesis to be tested is whether chronic back pain and certain psychiatric disorders may share a common risk factor, such as psychological stress.

Considering similar biological pathogenesis, the development of pain, depression and anxiety share the participation of neurotransmitters’ such as serotonin, norepinephrine, gamma-amino-butyric-acid, glutamate, adenosine, cannabinoids, and many other neuropeptides [15]. Functional magnetic resonance imaging (MRI) studies of subjects with chronic pain and depression (or anxiety) have shown common areas of brain activation. Additional mechanisms are related to the activation of the sympathetic nervous system, involvement of the hypothalamic-pituitary-axis, and downregulation of benzodiazepine receptors in the frontal cortex [5].

From the results shown in Tables 3 and 4, it becomes obvious that quality of life in patients with backache is affected due to pain interfering with patients’ daily activities and to patients’ psychological disturbance, anxiety and depressive symptoms. Pain may have a more negative effect on quality of life than psychological status. Table 3 shows that improvement of quality of life was strongly related to improvements of both pain and depression.

It was not unexpected that depression scores correlated negatively with each of SF-36 vitality and general health perception as this reflects some genuine depressive features—fatigue and negative self-perception. The same is also true in case of anxiety and vitality.

Lamé IE and his colleagues investigated pain cognitions and quality of life in a heterogeneous group of 1208 chronic pain patients. Patients with low back pain and multiple pain localizations experienced most functional limitations [16]. Pain catastrophizing turned out to be the single most important predictor of quality of life. Especially, social functioning, vitality, mental health, and general health were significantly associated with pain catastrophizing. In our study, anxiety scores were negatively correlated with each of social functioning, vitality, mental health, and physical functioning.

So backache interfering with patient’s different aspects of life, affecting his/her quality of life, including mental health, becomes associated with anxiety, phobia, of experiencing such pain while doing whatever activity “Pain catastrophizing.” Pain catastrophizing affects patient quality of life more and more. Depression increases secondary to the perception of impaired quality of life and strongly correlated with anxiety increase. While improving and when not experiencing pain in different situations, anxiety decreases, quality of life improves, and so does depression.

### Limitations

The present study had several limitations, of which the small number of patients was the most important.

## Conclusions

From the present study, we can reach some conclusions. First, spinal traction combined with medical treatment is effective for treating patients with chronic backache, and durations of treatment more than 4 weeks are not so much superior. Second, patients with chronic backache experience psychological disturbance in the form of anxiety and depressive symptoms which could worsen the patients’ conditions. Third, together with anxiety and depression, pain affects the quality of life of patients with chronic backache. Finally, further revisions of the usage of pain measurements that relate pain to patients’ activities, and not a general subjective measurement, should be considered.

## Abbreviations

HADS: Hospital anxiety and depression scale
SF-36: Short-Form 36 Health Survey
VAS: Visual analog scale
